# Supplementary material for: Evolution of the tuberculin skin test reveals generalisable Mtb-reactive T cell metaclones
Source: Nat Commun. 2026 Jan 21;17:1900. doi: 10.1038/s41467-026-68678-9 (PMC12923891; doi:10.1038/s41467-026-68678-9)
Supplement: Supplementary file 1 — Supplementary Information [file 41467_2026_68678_MOESM1_ESM.pdf]

Supplementary Information for

Evolution of the tuberculin skin test reveals generalisable *Mtb*-reactive T cell metaclones

Authors

Carolin T Turner[1†], Andreas Tiffeau-Mayer[1,2†], Joshua Rosenheim[1,14], Aneesh Chandran[1,15], Rishika Saxena[1], Ping Zhang[3], Jana Jiang[1,4], Michelle Berkeley[1], Flora Pang[1], Imran Uddin[1,16], Gayathri Nageswaran[1], Suzanne Byrne[1], Akshay Karthikeyan[1], Werner Smidt[5], Paul Ogongo[6, 17], Rachel Byng-Maddick[1], Santino Capocci[7,8], Marc Lipman[9,10], Heinke Kunst[11], Stefan Lozewicz[12], Veron Ramsuran[5,13], Gabriele Pollara[1], Julian C Knight[3], Alasdair Leslie[1,6], Benjamin M Chain[1], Mahdad Noursadeghi[1,8*]

[†] These authors contributed equally: Carolin T Turner, Andreas Tiffeau-Mayer

[*] Corresponding author. Email: m.noursadeghi@ucl.ac.uk

[1] Division of Infection and Immunity, University College London, London, United Kingdom; [2] Institute for the Physics of Living Systems, University College London, London, United Kingdom; [3] Centre for Human Genetics, University of Oxford, Oxford, United Kingdom; [4] Eye Center, Medical Center, Faculty of Medicine, University of Freiburg, Freiburg, Germany; [5] Centre for the AIDS Programme of Research in South Africa, Durban, South Africa; [6] Africa Health Research Institute, Durban, South Africa; [7] Department of Respiratory Medicine, University College London Hospitals NHS Foundation Trust, London, United Kingdom; [8] North Central London TB service, Whittington Health NHS Trust, London, United Kingdom; [9] Department of Respiratory Medicine, Royal Free London NHS Foundation Trust, London, United Kingdom; [10] UCL Respiratory, Division of Medicine, University College London, London, United Kingdom; [11] Queen Mary & Barts Health Tuberculosis Centre, Blizard Institute, Faculty of Medicine & Dentistry, Queen Mary University London, London, United Kingdom; [12] Department of Respiratory Medicine, North Middlesex University Hospital, London, United Kingdom; [13] School of Medicine, College of Health Sciences, University of KwaZulu-Natal, Durban, South Africa; [14] Present address: Department of Immunology and Microbiology, Immunology, University of Copenhagen, Copenhagen, Denmark; [15] Present address: Institute of Dentistry, Queen Mary University London, London, United Kingdom; [16] Present address: Cancer Institute, University College London, London, United Kingdom; [17] Present address: Division of Experimental Medicine, University of California San Francisco, San Francisco, United States.

Supplementary Information contents

Supplementary Table 1 3
Day 2 TST induration is correlated with T cell gene signatures and proxies for Mtb reactivity in day 7 TSTs. 3
Supplementary Figure 1 4
Consort diagram for application of samples in RNAseq and TCRseq analyses. 4
Supplementary Figure 2 5
Increased cell cycle activity in Day 7 TSTs. 5
Supplementary Figure 3 7
TCR repertoire diversity metrics. 7
Supplementary Figure 4 8
Abundance of published antigen-reactive CDR3 sequences. 8
Supplementary Figure 5 9
TCR alpha sequences associated with donor-unrestricted T cells are not enriched in the TST. 9
Supplementary Figure 6 10
Within- and cross-donor convergence of TCR repertoires. 10
Supplementary Figure 7 11
Expansion of private PPD-reactive TCRs in Day 7 TSTs. 12
Supplementary Figure 8 13
Selective expansion of PPD-reactive CDR3s between paired Day 2 and Day 7 TST samples. 13
Supplementary Figure 9 14
Published Mtb reactivity of class II-restricted metaclones. 14
Supplementary Figure 10 15
Effect of filtering on sensitivity and specificity of Gliph2 similarity clusters. 15
Supplementary Figure 11 16
Abundance of CDR3β metaclones in full repertoire of TCR sequences. 16
Supplementary Figure 12 17
Metaclones are more frequent in Day 7 TST compared to published Mtb-reactive CDR3 sequences. 17
Supplementary Figure 13 18
Mtb-reactive metaclones constitute a small proportion of the Day 7 TST repertoire. 18
Supplementary Figure 14 19
Publicity of Mtb-reactive TCRs in the Day 7 TST. 19
Supplementary Figure 15 20
Comparison of TST metrics between participants with presumed recent vs. remote exposure to TB. 20
References 22

Supplementary Table 1

	Day 2 TST	Day 7 TST	Blood
**Transcriptional modules**			
Module 'All T cells'	**ρ =0.32 FDR=2.2e-05**	**ρ =0.32 FDR=4.5e-04**	*NA*
Module 'CD4 T cells'	**ρ =0.31 FDR=3.6e-05**	**ρ =0.4 FDR=7.6e-06**	*NA*
Module 'CD8 T cells'	**ρ =0.47 FDR=1.1e-10**	**ρ =0.22 FDR=2.2e-02**	*NA*
Module 'NK cells'	**ρ =0.15 FDR=4.3e-02**	ρ =-0.03 FDR=7.3e-01	*NA*
Module 'Myeloid cells'	**ρ =0.45 FDR=3.5e-10**	ρ =0.12 FDR=2.6e-01	*NA*
Module 'Proliferation'	**ρ =-0.21 FDR=5.7e-03**	ρ =0.064 FDR=5.5e-01	*NA*
**TCR metrics**			
% metaclones	ρ =0.83 FDR=0.292	**ρ =0.22 FDR=0.036**	ρ =0.018 FDR=0.969
% TCRs with clone size >1	ρ =-0.43 FDR=0.803	ρ =0.061 FDR=0.525	ρ =0.71 FDR=0.209
% TCRs with clone size >2	ρ =-0.14 FDR=0.803	ρ =0.18 FDR=0.080	ρ =0.61 FDR=0.209
% TCRs with clone size >3	ρ =0.14 FDR=0.803	**ρ =0.26 FDR=0.016**	ρ =0.61 FDR=0.209
% TCRs with clone size >4	ρ =0.14 FDR=0.803	**ρ =0.31 FDR=0.004**	ρ =0.61 FDR=0.209

Day 2 TST induration is correlated with T cell gene signatures and proxies for Mtb reactivity in day 7 TSTs.

Correlation analysis of TST induration on day 2 with expression of gene signatures in TSTs from day 2 (n=184) or day 7 (n=135), and with percentage of β-chain TCR metrics in down-sampled repertoires from day 2 TST (n=6), day 7 TST (n=111) and blood (n=7). Two-sided Spearman correlation coefficients (ρ) and FDR-adjusted p-values are tabulated. Multiple testing correction was applied separately to correlation analysis of induration with transcriptional modules or with TCR metrics. Significant correlations (FDR<0.05) are highlighted in bold.

Supplementary Figure 1

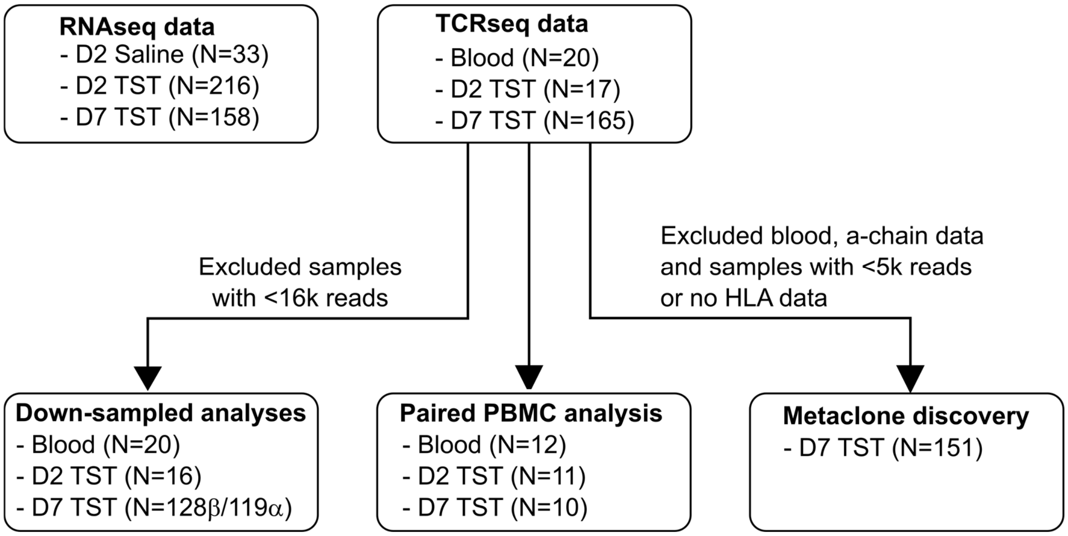

Consort diagram for application of samples in RNAseq and TCRseq analyses.

The data analysed in the present manuscript was derived from a cohort 216 individuals who underwent an intradermal tuberculin skin test in each arm and a separate cohort of 33 individuals who underwent an intradermal saline injection in one arm. In the TST cohort, total RNA from punch biopsies at the site of the TST were available from all 216 individuals at day 2, and 158 individuals at day 7. In the 'saline' cohort, total RNA from punch biopsies at the site of saline injection was available from all 33 individuals at day 2. Genome-wide transcriptional profiling by RNA sequencing was performed in all available RNA samples from these cohorts. TCR sequencing initially focussed on a subset of day 2 TST and paired blood samples. This was then extended to all day 7 TST samples.

Supplementary Figure 2

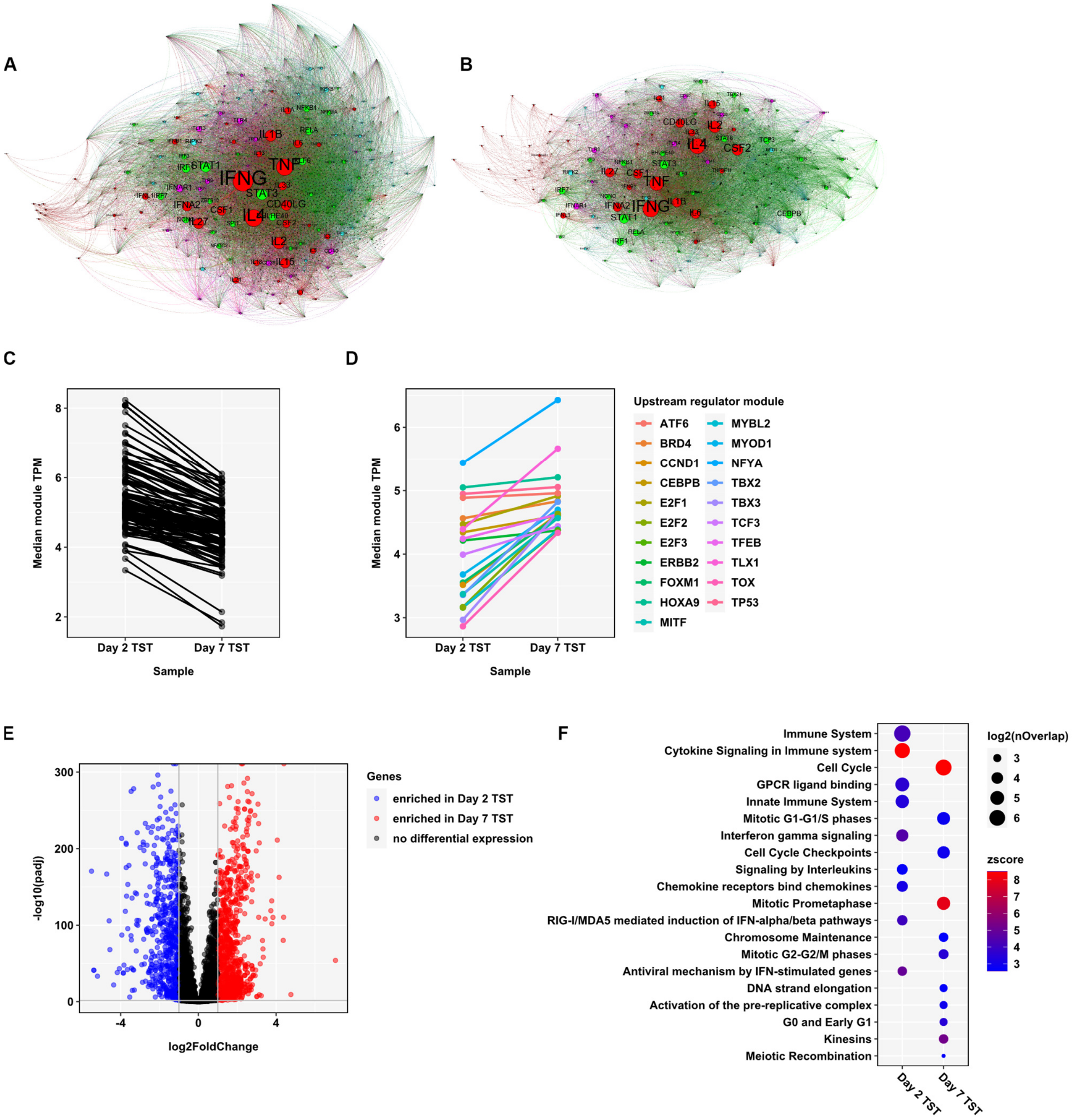

Increased cell cycle activity in Day 7 TSTs.

Bulk RNA sequencing of n=33 saline controls, n=216 Day 2 TST and n=158 Day 7 TST samples was used to define the TST response. **(A-B)** Network diagrams of statistically significant regulators (FDR <0.05; labelled nodes), predicted to act upstream of genes (black nodes) enriched in either Day 2 TSTs (A) or Day 7 TSTs (B) compared to saline-injected control skin. Upstream regulators were stratified by molecular function (different colour of nodes), with node size proportional to -log10 p value. Nodes were clustered using Force Atlas 2 algorithm in Gephi (version 0.9.4). **(C-D)** Statistically significant upstream regulators (FDR <0.05) were identified for genes enriched in the comparison of all TST samples (Day 2 and Day 7) vs saline controls. The mean TPM (transcript per million) expression of the target genes for each upstream regulator (= module score) was quantified in individual Day 2 and Day 7 TST samples, and the median of these module scores calculated per group. Upstream regulators were stratified by whether the median score decreased **(C)** or increased **(D)** significantly (adjusted p-value < 0.05) between Day 2 and Day 7, using unpaired, two-sided Wilcoxon tests with Benjamini-Hochberg correction for multiple testing. Each dot represents an upstream regulator module, showing the median score per group. In D, the legend lists the upstream regulator names, which are all associated with cell cycle activity. **(E)** Volcano plot showing statistical significance against quantitative gene expression differences between Day 2 and Day 7 TST samples, amongst 4150 integrated genes that are

enriched in either Day 2 or Day 7 TSTs compared to saline controls. Genes highlighted in red and blue are upregulated on Day 2 (n=608) and Day 7 (n=1003), respectively, with an FDR <0.05 and fold change ≥2. **(F)** Enrichment of Reactome pathways among the genes differentially expressed between Day 2 and Day 7 TSTs, as identified in E. Node size indicates number of the differentially expressed genes that are associated with each pathway. Node colour represents the statistical enrichment Z score.

Supplementary Figure 3

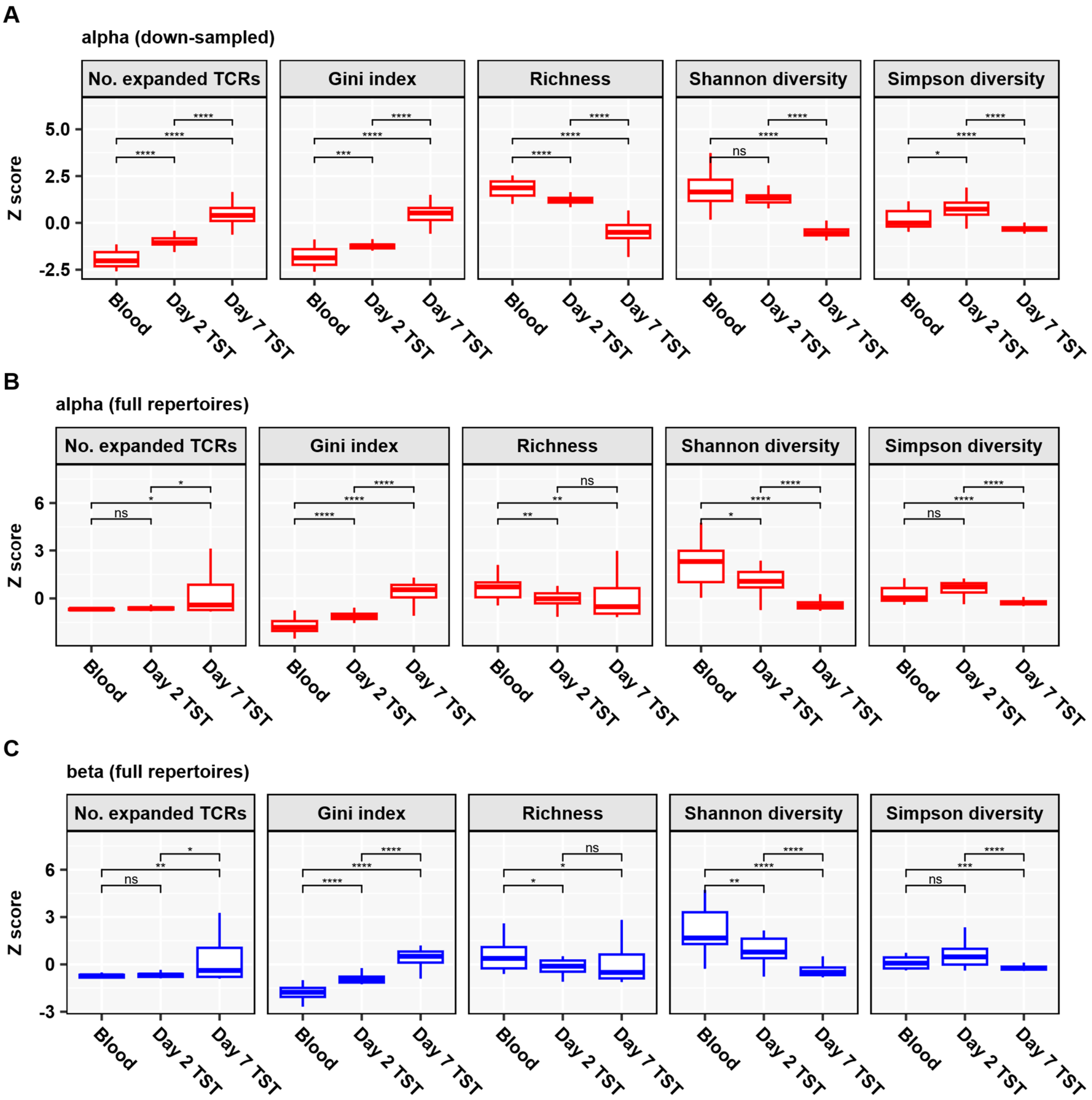

TCR repertoire diversity metrics.

Data are shown as Z-score values scaled across all samples, with boxplots depicting median and inter-quartile range (IQR), while outlier data points (more than 1.5*IQR beyond the box hinges) are shown as dots. Statistical significance was assessed with unpaired, two-sided Wilcoxon tests and corrected for multiple testing (ns FDR>0.05, * FDR<0.05, ** FDR<0.01, *** FDR<0.01, **** FDR<0.0001). No. expanded TCRs = number of TCR sequences present more than once. **(A)** Diversity metrics calculated after individual alpha chain bulk TCR repertoires were down-sampled to 16,000 total TCRs (n=20 Blood, n=16 Day 2 TST, n=119 Day 7 TST). **(B-C)** Diversity metrics calculated in full repertoires (n=20 Blood, n=17 Day 2 TST, n=165 Day 7 TST), using either alpha **(B)** or beta **(C)** chain sequences.

.

Supplementary Figure 4

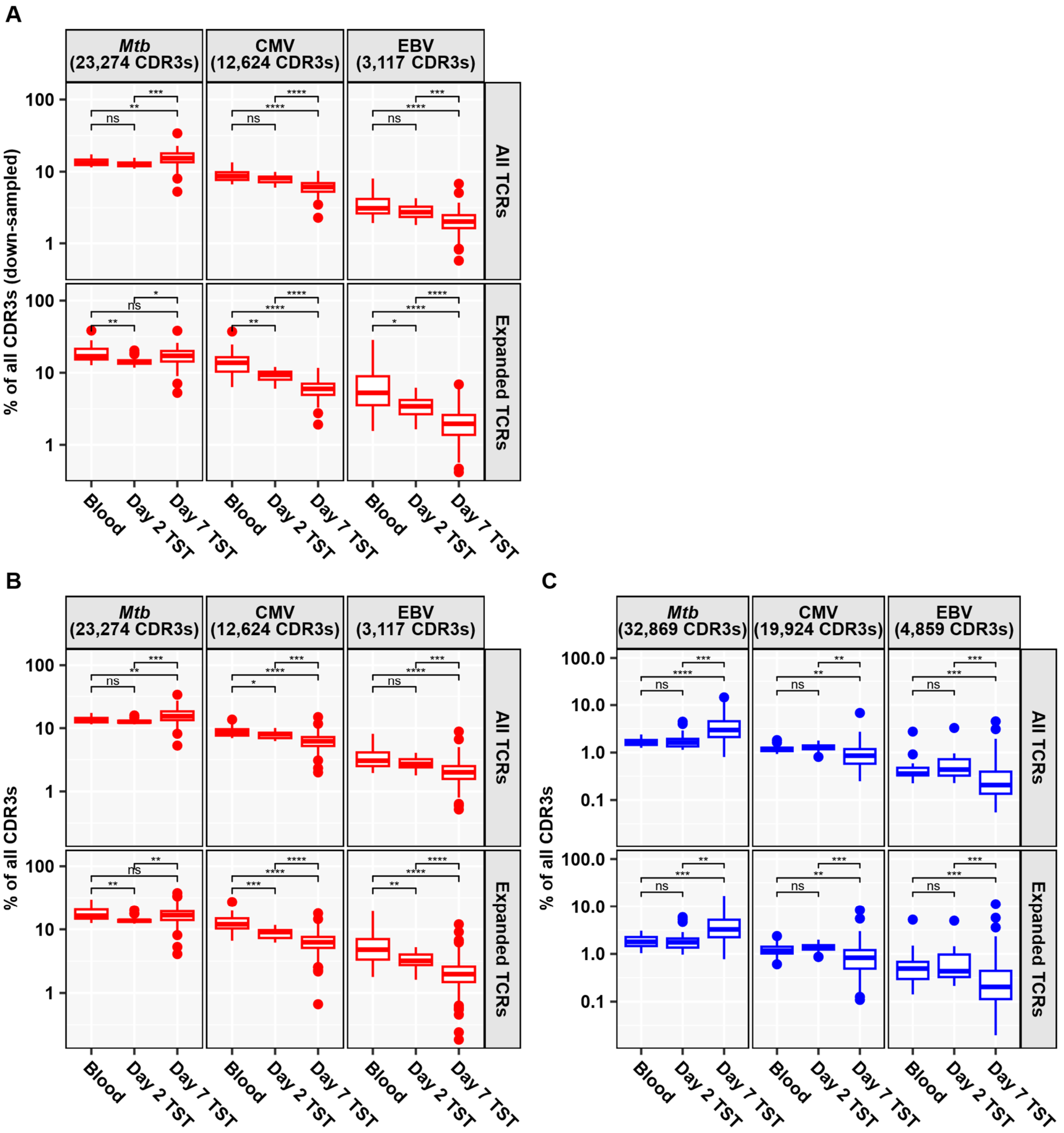

Abundance of published antigen-reactive CDR3 sequences.

Antigen-reactive CDR3s (specific for *Mtb*, CMV or EBV) were collated from VDJdb and McPAS databases as well as from Musvosvi et al.[1], and their abundance in Blood and TST repertoires is shown as percentage of all TCRs or of all expanded TCRs (present more than once). The number of distinct published antigen-reactive CDR3s available to assess enrichment of antigen reactivity is indicated. Boxplots display median and inter-quartile range (IQR), with outlier data points (more than 1.5*IQR beyond the box hinges) shown as dots. Statistical significance was assessed with unpaired, two-sided Wilcoxon tests and corrected for multiple testing (ns FDR>0.05, * FDR<0.05, ** FDR<0.01, *** FDR<0.01, **** FDR<0.0001). **(A)** Abundance calculated after individual alpha chain bulk TCR repertoires were down-sampled to 16,000 TCRs (n=20 Blood, n=16 Day 2 TST, n=119 Day 7 TST). **(B-C)** Abundance calculated in full repertoires (n=20 Blood, n=17 Day 2 TST, n=165 Day 7 TST), using either alpha **(B)** or beta **(C)** chain sequences.

Supplementary Figure 5

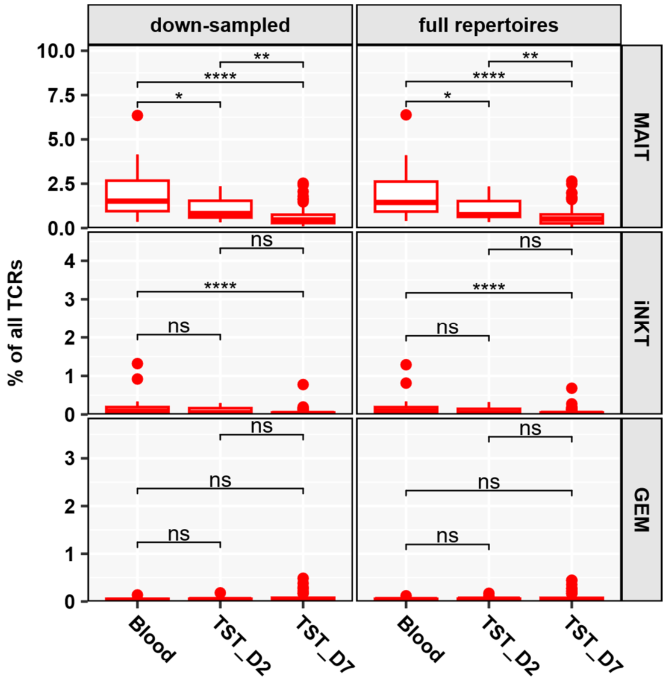

TCR alpha sequences associated with donor-unrestricted T cells are not enriched in the TST. Percentage of alpha chain sequences associated with mucosal-associated invariant T (MAIT), invariant natural killer T (iNKT) and germline-encoded mycolyl lipid-reactive (GEM) T cells in blood, day 2 and day 7 TST TCR repertoires. Abundance was calculated in full repertoires (n=20 Blood, n=17 Day 2 TST, n=165 Day 7 TST) and after individual alpha chain bulk TCR repertoires were down-sampled to 16,000 total TCRs (n=20 Blood, n=16 Day 2 TST, n=119 Day 7 TST). Data are shown as boxplots depicting median and interquartile range (IQR), while outlier data points (more than 1.5*IQR beyond the box hinges) are shown as dots. Statistical significance was assessed with unpaired, two-sided Wilcoxon tests and corrected for multiple testing (ns FDR>0.05, * FDR<0.05, ** FDR<0.01, **** FDR<0.0001).

Supplementary Figure 6

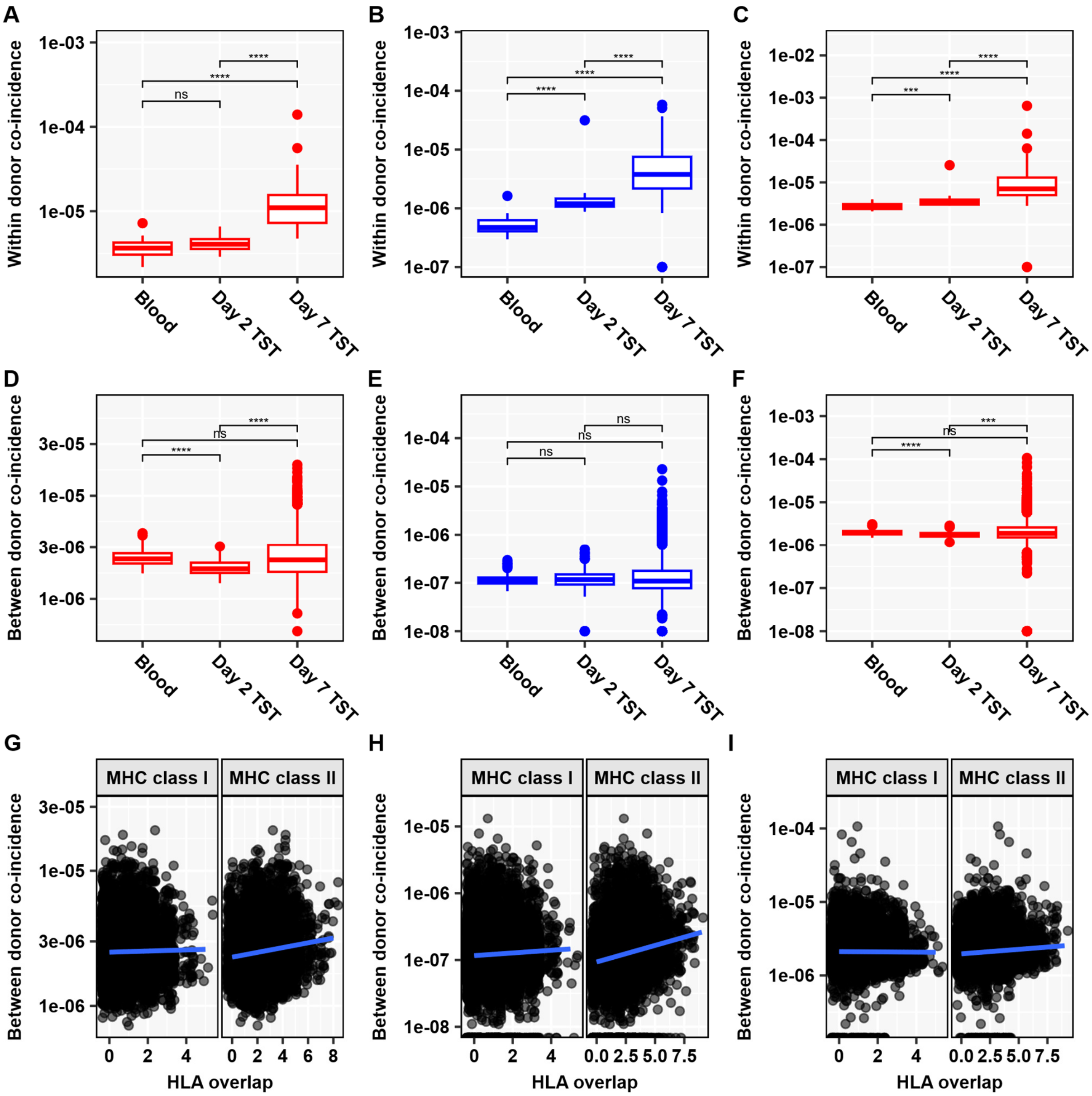

Within- and cross-donor convergence of TCR repertoires.

(A-C) Within-donor convergence of different TCR nucleotide sequences onto identical amino acid sequences in down-sampled alpha **(A)**, full beta **(B)** or full alpha **(C)** repertoires. The number of samples in down-sampled alpha repertoires **(A)** is n=20 Blood, n=16 Day 2 TST and n=119 Day 7 TST. The number of samples in full repertoires **(B-C)** is n=20 Blood, n=17 Day 2 TST and n=165 Day 7 TST. **(D-F)** Cross-donor TCR convergence, calculated in any two individuals, in down-sampled alpha **(D)**, full beta **(E)** or full alpha **(F)** repertoires. The number of pairwise comparisons for down-sampled alpha repertoires **(D)** is n=190 Blood, n=120 Day 2 TST and n=7021 Day 7 TST. The number of pairwise comparisons for full repertoires **(E-F)** is n=190 Blood, n=136 Day 2 TST and n=13530 Day 7 TST. **(G-I)** Cross-donor TCR convergence in Day 7 TSTs, stratified by the number of class 1 or class 2 HLA alleles shared between any two individuals, using down-sampled alpha **(G)**, full beta **(H)** or full alpha **(I)** repertoires.

The boxplots in A-F depict median and inter-quartile range (IQR) for each group, with outlier data points (more than 1.5*IQR beyond the box hinges) shown as dots. Statistical significance was assessed with unpaired, two-sided Wilcoxon tests and corrected for multiple testing (ns FDR>0.05, *** FDR<0.001, **** FDR<0.0001). In G-I, each dot represents a pairwise comparison, with linear regression line shown in blue.

Supplementary Figure 7

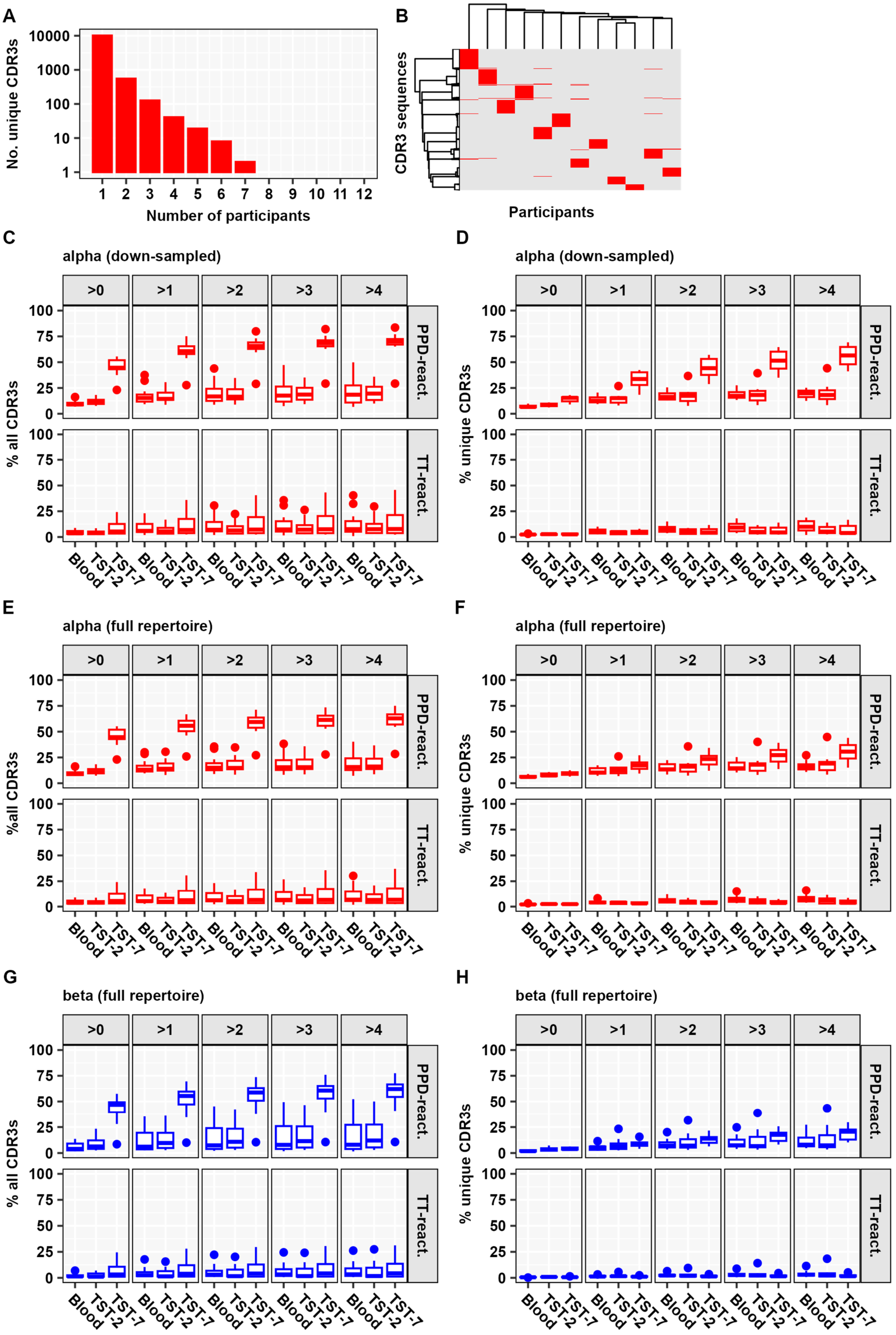

Expansion of private PPD-reactive TCRs in Day 7 TSTs.

PBMCs from a subset of participants (n=12) were stimulated in vitro with one of 10 µg/ml purified protein derivative of *Mtb* (PPD), 100 µg/ml tetanus toxoid (TT), or control media for 6 days. Antigen-reactive CDR3s were identified by bulk TCR sequencing as CDR3s with ≥8-fold increase in count in PPD or TT cultures, but not unstimulated control cultures, compared to ex vivo blood from the same individual. **(A)** Histogram showing the number of unique in vitro PPD-reactive alpha chain CDR3s shared by different numbers of participants, demonstrating that the majority of PPD-reactive CDR3s are private (= found in only single participants). **(B)** Heatmap of unique in vitro PPD-reactive alpha chain CDR3s per participant. The dendrogram depicts Ward D2 linkage clustering. **(C-D)** Abundance of private PPD- or TT-reactive CDR3 sequences in down-sampled alpha chain repertoires from blood and TSTs, stratified by clone size (= TCR count). Abundance was quantified as percentage of total **(C)** or unique **(D)** CDR3s. **E-F.** Abundance of private PPD- or TT-reactive CDR3 sequences in full beta chain repertoires from blood and TSTs; quantified as percentage of total **(E)** or unique **(F)** CDR3s. **(G-H)** Abundance of private PPD- or TT-reactive CDR3 sequences in full alpha chain repertoires from blood and TSTs; quantified as percentage of total **(G)** or unique **(H)** CDR3s. The boxplots in C-H display median and inter-quartile range (IQR), with outlier data points (more then 1.5*IQR beyond the box hinges) shown as dots. Only participants used for paired in vitro stimulation experiments were used in this analysis (n=12 Blood, n=11 Day 2 TST, n=10 Day 7 TST).

Supplementary Figure 8

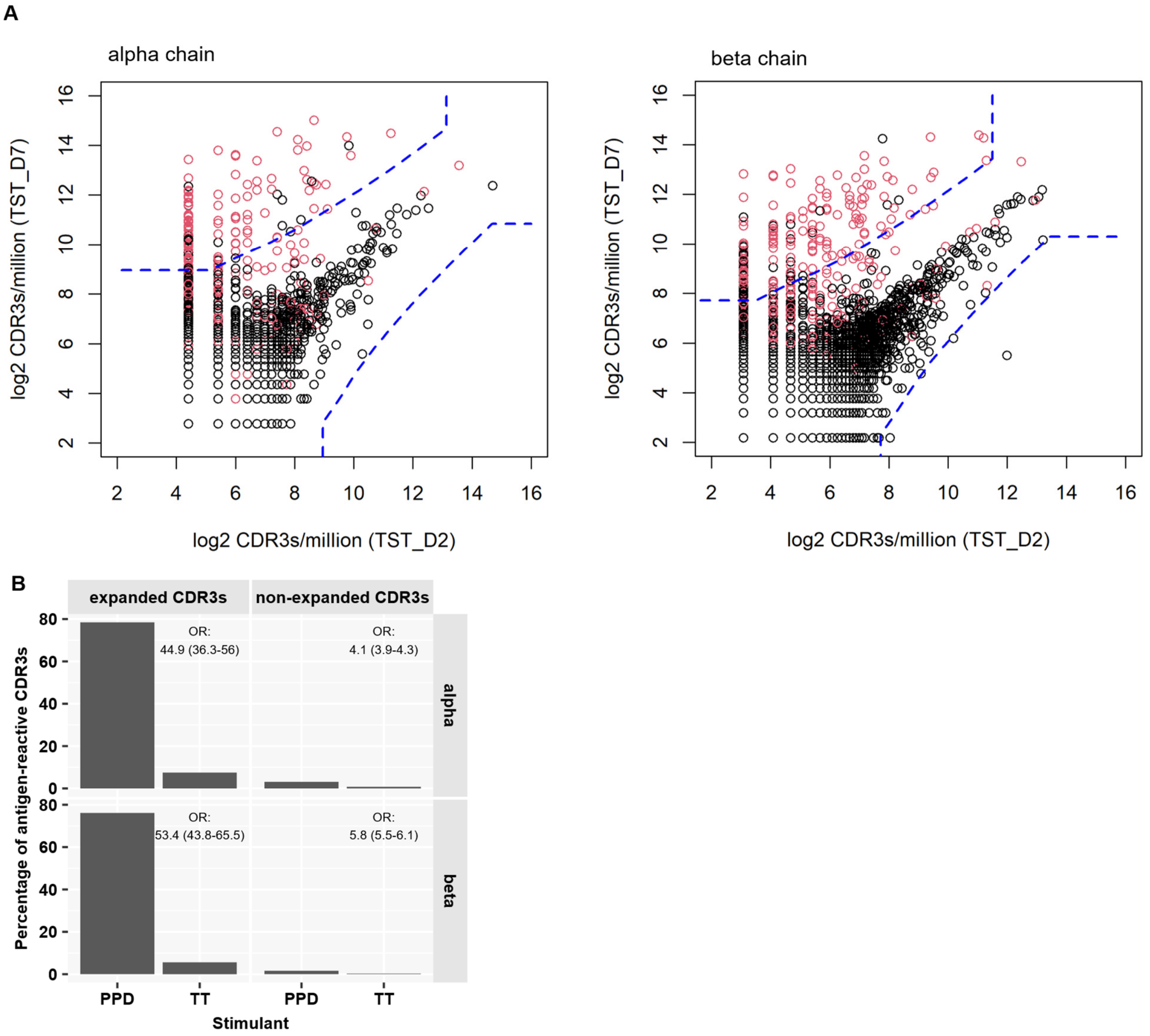

Selective expansion of PPD-reactive CDR3s between paired Day 2 and Day 7 TST samples.

(A) A representative example of the pairwise comparison between Day 2 and Day 7 TST TCRs from the same individual (donor LAT01). Each point is an individual CDR3 sequence, with its abundance on Day 7 plotted versus its abundance on Day 2. CDR3s absent in one of the paired samples were replaced with the median CDR3 count of that sample, and all CDR3 counts were then log-normalised (log2 counts/million). Dots in red represent PPD-reactive CDR3s, defined with in vitro stimulation experiments (see Figures 3 and S7). The dashed blue lines indicate the significance thresholds ($p < 0.0001$) for a Poisson distribution of counts, with CDR3s that fall to the left and above the dashed line considered expanded on Day 7. **(B)** Expanded and non-expanded CDR3s between paired Day 2 and Day 7 TSTs were integrated across n=10 individuals, stratified by chain, and their PPD and TT reactivity assessed using antigen-reactive CDR3s defined after in vitro stimulation (see Figures 3 and S7). Odds ratios (OR) with 95% confidence intervals were calculated to quantify enrichment of PPD vs TT reactivity amongst expanded and non-expanded clones.

Supplementary Figure 9
A

Metaclone description	Matching CDR3	VDJdb entry
Index 100<br>Consensus CDR3: CSARGQGNEQFF<br>V: TRBV20-1	CSARGQGNEQYF	V: TRBV20-1*01<br>Species: *M.tuberculosis*<br>Protein: PPE33<br>Epitope: PPQIAANRSQLISLV
Index 47<br>Consensus CDR3: CSARASGGEAKNIQYF<br>V: TRBV20-1	CSARASGGEAKNIQYF,<br>CSARAGGGEAKNIQYF	V: TRBV20-1*01<br>Species: *M.tuberculosis*<br>Protein: Rv3874<br>Epitope: AAVVRFQEAANKQKQ
Index 12<br>Consensus CDR3: CASSLIENTEAFF<br>V: TRBV12-4	CASSLIENTEAFF	V: TRBV12-3*01<br>Species: *M.tuberculosis*<br>Protein: Rv3804c<br>Epitope: PSPSMGRDIKVQFQS

B

Metaclone 100:
Most abundant in *Mtb*-reactive T cells from Musvosvi et al. 2023
1 member CDR3 annotated as reactive to *Mtb* PPE33 in VDJdb

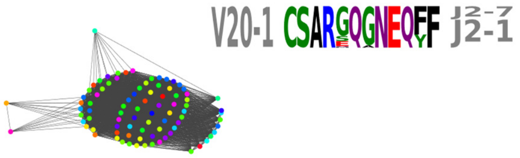

Metaclone 156:
Most public in *Mtb*-reactive T cells from Musvosvi et al. 2023

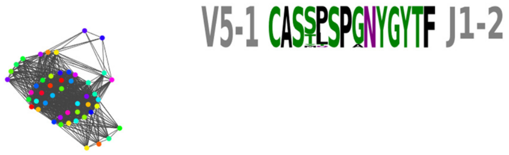

Metaclone 47:
2 member CDR3s annotated as reactive to *Mtb* Rv3874 in VDJdb

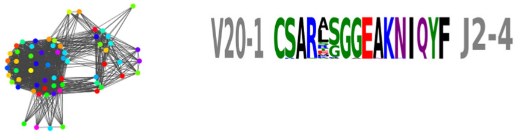

Metaclone 12:
1 member CDR3 annotated as reactive to *Mtb* Rv3804c in VDJdb

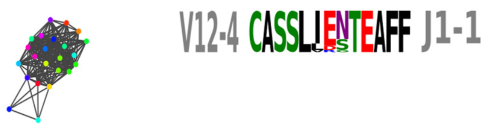

Published Mtb reactivity of class II-restricted metaclones.
(A) CDR3 sequences included in class II-restricted metaclones (n=1392) were compared against CDR3 β sequences annotated in VDJdb with MHC class "MHCII", and antigen species "*M.tuberculosis*" or "*Mtb*" (n=30). The middle column shows the matching CDR3; the left-hand column shows the description of the metaclone that the CDR3 is a member of; the right-hand column shows VDJdb annotations for the CDR3.
(**B**) Adjacency graphs and sequence logos for metaclones with *Mtb* CDR3 matches in VDJdb, and for metaclones that are most abundant (matching 47 out of 21,212 cells with β-chain TCR data) or most public (found in 14 out of 70 participants) in single cell TCRseq data from *Mtb*-reactive T cells described by Musvosvi et al.[1]. Each node in the adjacency graph represents a single TCR stratified by colour into distinct donors from the Day 7 TST discovery dataset.

Supplementary Figure 10
A

	Number clusters	Number unique CDR3s
Gliph2 + Filter 1	1837	6546
Gliph2 + Filter 1 + Filter 2 (p<0.05)	1342	5022
Gliph2 + Filter 1 + Filter 2 (FDR <0.1)	106	476
Gliph2 + Filter 2 (FDR <0.1)	128	476

B

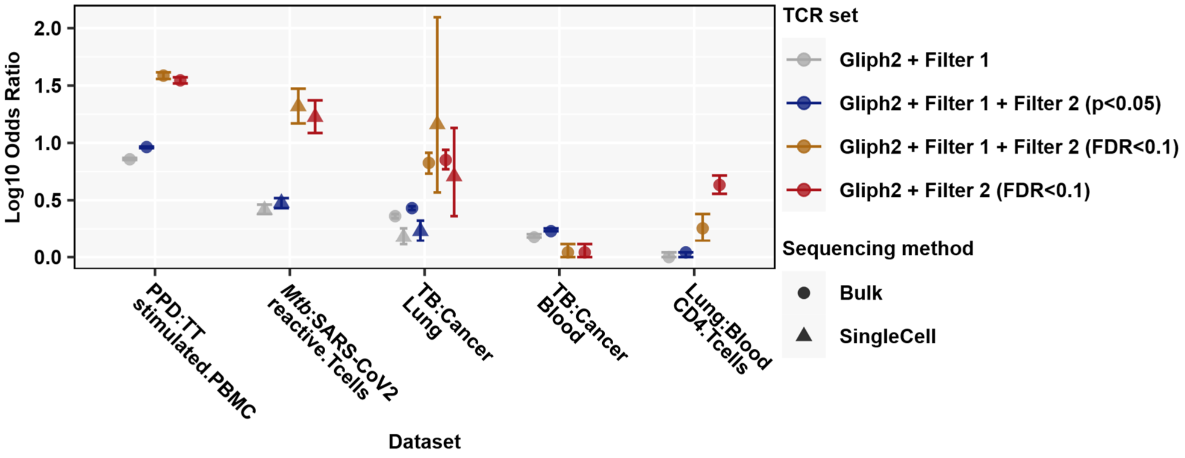

Effect of filtering on sensitivity and specificity of Gliph2 similarity clusters.

Gliph2 similarity clusters were derived from expanded (TCR count >1) day 7 TST β-chain repertoires (122,253 TCR clones, 151 individuals). Gliph2 clusters containing CDR3s from ≥4 participants were selected for analysis. Filter 1 retained clusters with ≥3 unique CDR3s, which had a Fisher_score, vb_score and length_score ≤0.05 each. Filter 2 retained clusters with a Fisher's exact test for HLA class II association of p<0.05 or FDR<0.1. **(A)** Number of Gliph2 clusters and number of unique day 7 TST CDR3s selected in each analysis iteration. **(B)** Relative enrichment of CDR3s clustered by Gliph2 and filtered by different criteria in multiple external data sets, showing odds ratio (OR) point estimates with 95% confidence intervals for the pairwise comparisons indicated. See Figure 5 in the main manuscript for a detailed description of the datasets. Results for 'Gliph2 + Filter 2 (FDR<0.1)' are replicated in Figure 5 in the main manuscript as 'Gliph2-matching CDR3s'.

Supplementary Figure 11

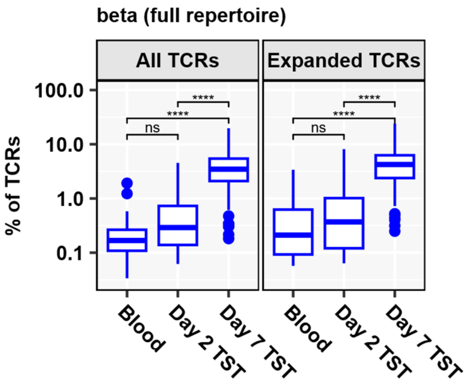

Abundance of CDR3β metaclones in full repertoire of TCR sequences.

In the full repertoire of TCRβ chain sequences, abundance of HLA class 2-restricted metaclones in individual blood or TST TCR repertoires, shown as percentage of all TCRs or of all expanded TCRs (present more than once). N=20 Blood, N=17 Day 2 TST, N=165 Day 7 TST. Boxplots display median and inter-quartile range (IQR), with outlier data points (more than 1.5*IQR beyond the box hinges) shown as dots. Statistical significance was assessed with unpaired, two-sided Wilcoxon tests and corrected for multiple testing (ns FDR>0.05, **** FDR<0.0001).

Supplementary Figure 12

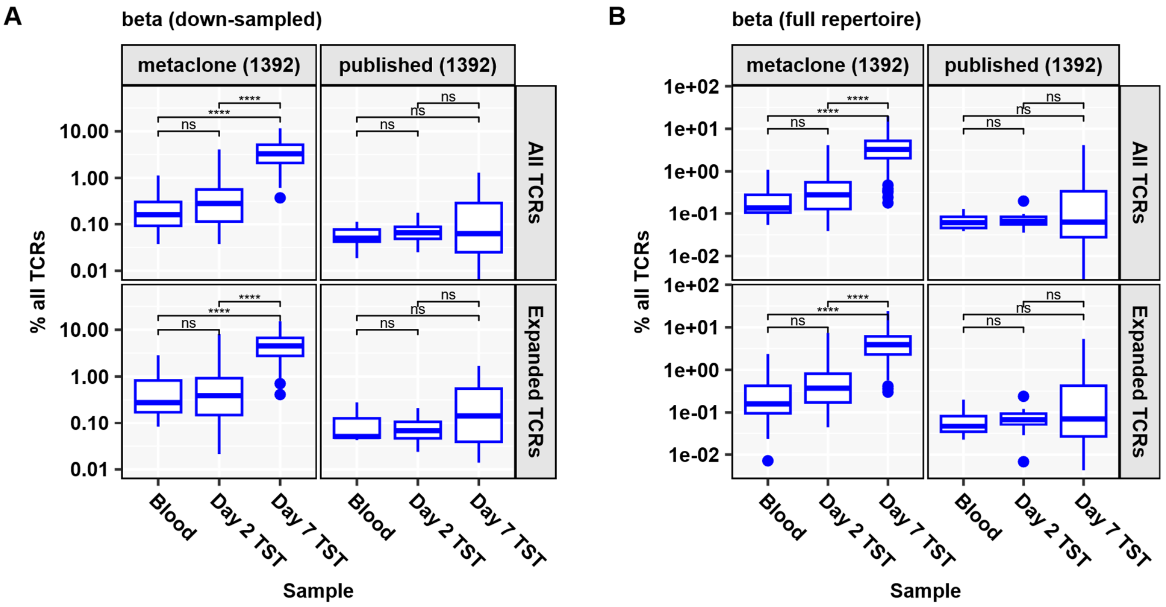

Metaclones are more frequent in Day 7 TST compared to published Mtb-reactive CDR3 sequences.

Abundance of HLA class II restricted metaclone CDR3s and equal numbers of randomly sampled published *Mtb*-reactive CDR3s in individual blood and TST repertoires, shown as percentage of all TCRs or of all expanded TCRs (present more than once). **(A)** Blood and TST repertoires were down-sampled to 16,000 β chain TCRs each from N=20 Blood, N=16 Day 2 TST, N=128 Day 7 TST samples. **(B)** Full β chain repertoires were used (N=20 Blood, N=17 Day 2 TST, N=165 Day 7 TST). Boxplots display median and inter-quartile range (IQR), with outlier data points (more than 1.5*IQR beyond the box hinges) shown as dots. Statistical significance was assessed with unpaired, two-sided Wilcoxon tests and corrected for multiple testing (ns FDR>0.05, * FDR<0.05, **** FDR<0.0001).

Supplementary Figure 13

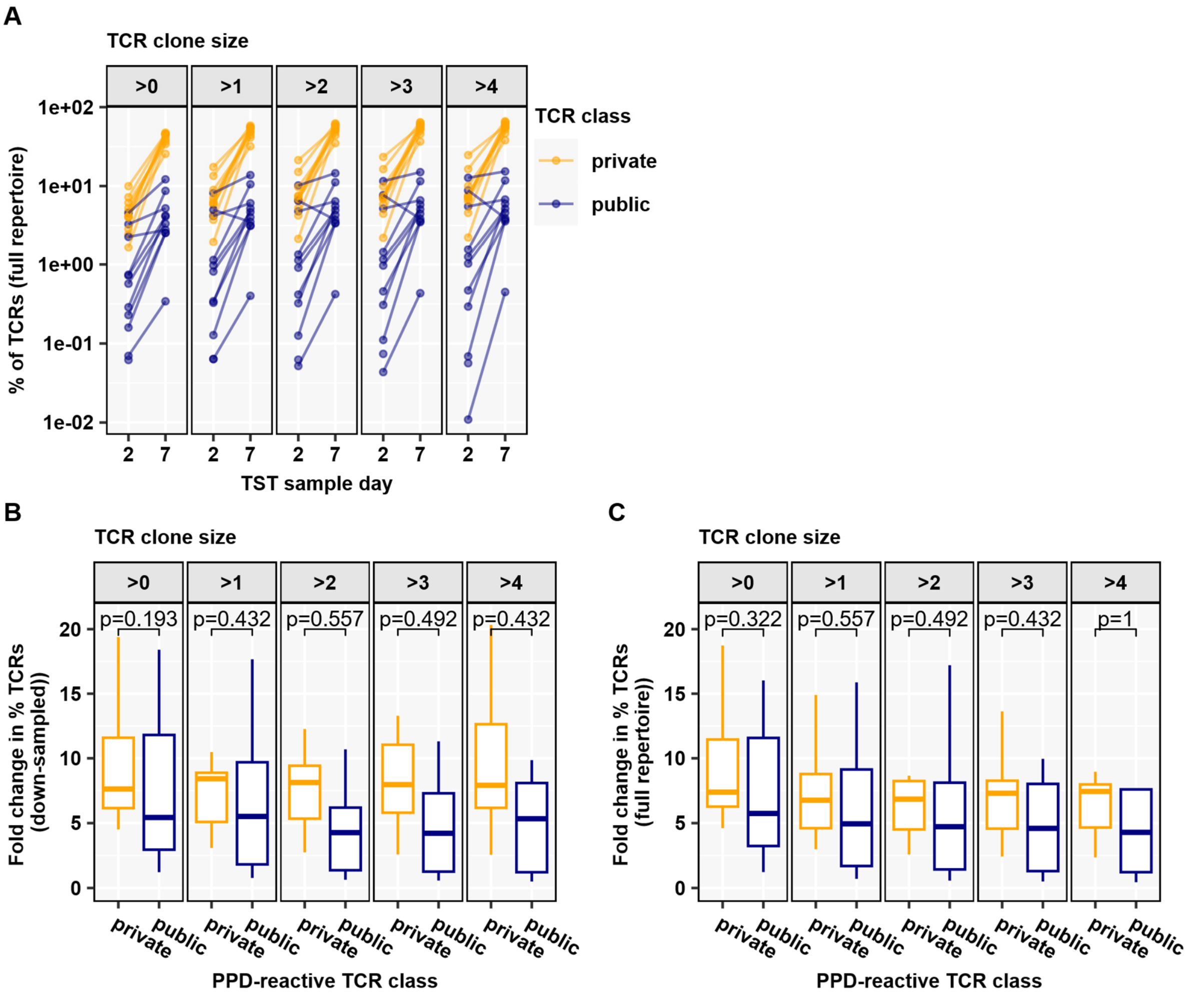

Mtb-reactive metaclones constitute a small proportion of the Day 7 TST repertoire.

(A) In the full repertoire of β chain TCR sequences, abundance of 'public' and 'private' *Mtb*-reactive CDR3s in the same individual was quantified as percentage of all Day 2 or all Day 7 TST TCRs, stratified by clone size (= TCR count). β–chain TCRs were classified as 'public' if they matched a Metaclonotypist metaclone, or else as 'private' if they matched a private PPD-reactive CDR3 sequence identified from ex vivo stimulated PBMC from the same individual (see Figure 3). CDR3s matching both a metaclone and private PPD-reactive CDR3 were classed as 'public'. This analysis was restricted to individuals with paired in vitro stimulation experiments (n=11 Day 2 TST, n=10 Day 7 TST). **(B-C)** Fold-change in private and public CDR3s in day 7 compared to day 2 TSTs stratified by clone size, using repertoires down-sampled to 16,000 TCRs each **(B)** or full repertoires **(C)**. Box and whisker plots represent median, interquartile range and 1.5 IQR limits for N=10 individuals. P values shown for paired, two-sided Wilcoxon tests for each analysis stratified clone size.

Supplementary Figure 14

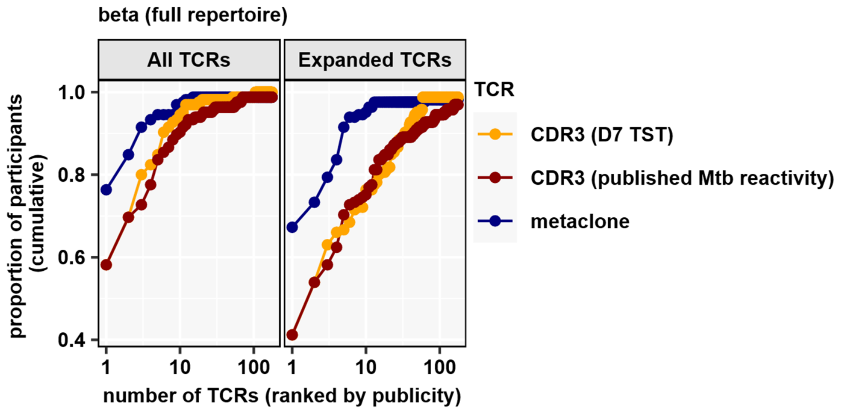

Publicity of Mtb-reactive TCRs in the Day 7 TST.

In the full repertoire of TCRβ chain sequences, HLA class II-restricted metaclones (blue), CDR3s with published *Mtb* reactivity (red), and CDR3s present in Day 7 TSTs (yellow) were each ranked by their publicity across all available Day 7 TST repertoires (n=165) and plotted against the cumulative proportion of participants expressing the TCR. Presence of TCRs was assessed using either all TCR sequences in each sample or only expanded TCRs (present more than once).

Supplementary Figure 15

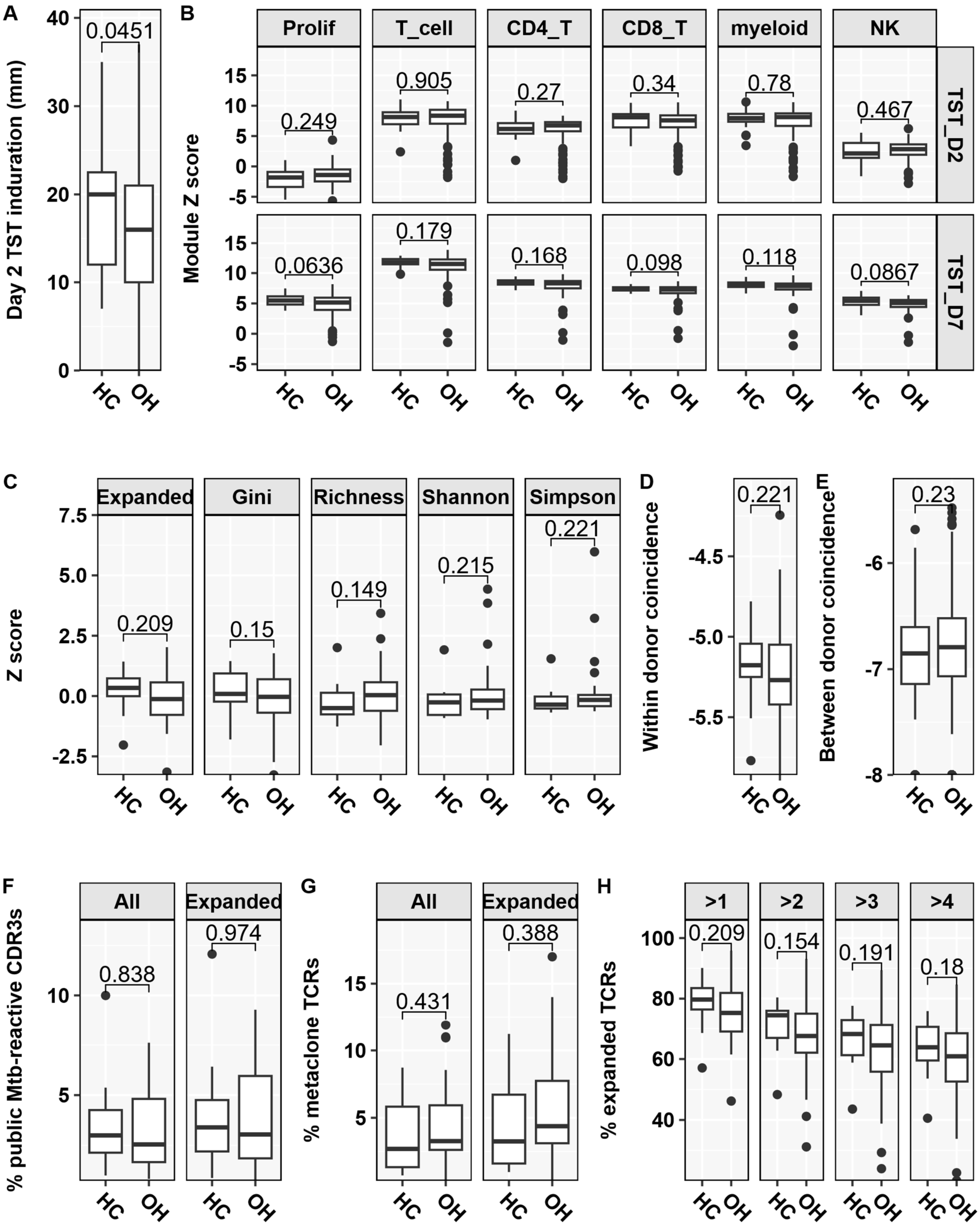

Comparison of TST metrics between participants with presumed recent vs. remote exposure to TB. Household contacts (HC) of active TB index cases were defined as recently exposed to TB, and participants whose immunological memory to *Mtb* was identified via routine occupational health (OH) screening were defined as remotely exposed to TB. **(A)** Day 2 TST induration (n=27 HC vs. n=86 OH). **(B)** Expression of cellular proliferation and cell type-specific modules in Day 2 and Day 7 TST bulk RNAseq data (D2: n=30 HC vs. n=100 OH; D7: n=27 HC vs. n=65 OH). **(C-E)** Diversity metrics **(C)**, within donor coincidence **(D)** and between donor coincidence **(E)** in down-sampled Day 7 TST bulk TCRseq beta chain data (n=17 HC vs. n=43

OH, resulting in n=136 pairwise HC and n=903 pairwise OC comparisons to calculate between donor coincidence). (**F-H)** Proxies for *Mtb*-reactivity in down-sampled Day 7 TST bulk TCRseq beta chain data (n=17 HC vs. n=43 OH), including percentage of public *Mtb*-reactive CDR3 sequences **(F)** and metaclone TCRs **(G)** amongst all or expanded (clone size >1) TCR sequences, and percentage of expanded TCRs, stratified by clone size (>1, >2, >3, >4) **(H)**. Statistical significance was assessed with unpaired, two-sided Wilcoxon tests.

References

1. Musvosvi M, Huang H, Wang C, Xia Q, Rozot V, Krishnan A, Acs P, Cheruku A, Obermoser G, Leslie A, Behar SM, Hanekom WA, Bilek N, Fisher M, Kaufmann SHE, Walzl G, Hatherill M, Davis MM, Scriba TJ. T cell receptor repertoires associated with control and disease progression following Mycobacterium tuberculosis infection. ***Nat Med***. 2023;29(1):258-269. doi:10.1038/s41591-022-02110-9.
