# Supplementary material for: Evolution of the tuberculin skin test reveals generalisable Mtb-reactive T cell metaclones
Source: Nat Commun. 2026 Jan 21;17:1900. doi: 10.1038/s41467-026-68678-9 (PMC12923891; doi:10.1038/s41467-026-68678-9)
Supplement: Supplementary file 2 — Description of Additional Supplementary Files [file 41467_2026_68678_MOESM2_ESM.pdf]

Description of Additional Supplementary Files

Title: **Supplementary Data 1**
Description: Sample metadata for RNAseq and TCRseq data from healthy volunteers with immunological memory to *Mycobacterium tuberculosis*, including skin-derived (saline, day 2 or day 7 TST) and blood-derived samples (blood, PBMC).

Title: **Supplementary Data 2**
Description: Composition of gene signatures for proliferation (CCND1) and cell types.

Title: **Supplementary Data 3**
Description: Published CDR3 sequences with reactivity to Mtb, CMV or EBV.

Title: **Supplementary Data 4**
Description**:** HLA genotype imputations from the Michigan Imputation Server. Column description: UIN = unique identifier for the sample; HLA = HLA allele being imputed; INFO = imputation quality metrics, including minor allele frequency (MAF) in the reference panel and imputation accuracy (R2); genotype = imputed genotype, shown as two phased alleles, where 1|1 indicates homozygosity and 1|0 and 0|1 indicate heterozygosity; estimated.pp_0.1 = posterior probability that the genotype is heterozygous; estimated.pp_1.1 = posterior probability that the genotype is homozygous.

Title: **Supplementary Data 5**
Description: HLA class II associated beta-chain metaclones, identified by Metaclonotypist from day 7 TST samples. Column description: index = re-numbered identifier for significant TCR cluster (metaclone); cluster = identifier for the TCR cluster (metaclone); hla = HLA allele tested for association; hla_frequency = total number of individuals with the specified HLA allele; count_allele = number of individuals within the cluster that have the specified HLA allele; total_allele = total number of individuals in the cluster; count_other = number of individuals not part of the cluster with the specified HLA allele; total_other = number of individuals not part of the cluster; pvalue = unadjusted p-value from a Fisher's exact test assessing the association between the cluster and the HLA allele; odds_ratio = odds ratio quantifying the strength of the association between the cluster and the HLA allele; significant = Bonferroni-Hochberg adjusted pvalue <0.1; Vs = V gene(s) associated with TCRs included in the TCR cluster (metaclone); consensus = consensus CDR3 amino acid sequences of TCRs included in the TCR cluster (metaclone); regex = regular expression for the CDR3 amino acid sequences included in the TCR cluster (metaclone); CDR3s = CDR3 amino acid sequences included in the TCR cluster.

Title: **Supplementary Data 6**
Description: HLA class I associated beta-chain metaclones, identified by Metaclonotypist from day 7 TST samples. Column description: index = re-numbered identifier for significant TCR cluster (metaclone); cluster = identifier for the TCR cluster (metaclone); hla = HLA allele tested for association; hla_frequency = total number of individuals with the specified HLA allele; count_allele = number of individuals within the cluster that have the specified HLA allele; total_allele = total number of individuals in the cluster; count_other = number of individuals not part of the cluster with the specified HLA allele; total_other = number of individuals not part of the cluster; pvalue = unadjusted p-value from a Fisher's exact test assessing the association between the cluster and the HLA allele; odds_ratio = odds ratio quantifying the strength of the association between the cluster and the HLA allele; significant = Bonferroni-Hochberg adjusted pvalue <0.1; Vs = V gene(s) associated with TCRs included in the TCR cluster (metaclone); consensus = consensus CDR3 amino acid sequences of TCRs included in the TCR cluster (metaclone); regex = regular expression for the CDR3 amino acid sequences included in the TCR cluster (metaclone); CDR3s = CDR3 amino acid sequences included in the TCR cluster.

Title: **Supplementary Data 7**
Description: HLA class II associated beta-chain GLIPH2 clusters, identified from day 7 TST samples. Column description: index = re-numbered identifier for significant GLIPH2 cluster; cluster = identifier for the GLIPH2 cluster; hla = HLA allele tested for association; hla_frequency = total number of individuals with the specified HLA allele; count_allele = number of individuals within the cluster that have the specified HLA allele; total_allele = total number of individuals in the cluster; count_other = number of individuals not part of the cluster with the specified HLA allele; total_other = number of individuals not part of the cluster; pvalue = unadjusted p-value from a Fisher's exact test assessing the association between the cluster and the HLA allele; odds_ratio = odds ratio quantifying the strength of the association between the cluster and the HLA allele; significant = Bonferroni-Hochberg adjusted pvalue <0.1; pattern = regular expression for the GLIPH2 similarity pattern of the CDR3 amino acid sequences included in the TCR cluster; CDR3s = CDR3 amino acid sequences included in the TCR cluster.

Title: **Supplementary Data 8**
Description: HLA class I associated beta-chain GLIPH2 clusters, identified from day 7 TST samples. Column description: index = re-numbered identifier for significant GLIPH2 cluster; cluster = identifier for the GLIPH2 cluster; hla = HLA allele tested for association; hla_frequency = total number of individuals with the specified HLA allele; count_allele = number of individuals within the cluster that have the specified HLA allele; total_allele = total number of individuals in the cluster; count_other = number of individuals not part of the cluster with the specified HLA allele; total_other = number of individuals not part of the cluster; pvalue = unadjusted p-value from a Fisher's exact test assessing the association between the cluster and the HLA allele; odds_ratio = odds ratio quantifying the strength of the association between the cluster and the HLA allele; significant = Bonferroni-Hochberg adjusted pvalue <0.1; pattern = regular expression for the GLIPH2 similarity pattern of the CDR3 amino acid sequences included in the TCR cluster; CDR3s = CDR3 amino acid sequences included in the TCR cluster.

Title: **Supplementary Data 9**
Description: Sample metadata for TCRseq data from TB patients and cancer controls, used for validation analyses of TST-derived metaclones, as shown in Figure 5 of the manuscript, and including lung tissue, whole blood and sorted CD4 T cells from lung and blood.
