# Supplementary material for: Evolution of the tuberculin skin test reveals generalisable Mtb-reactive T cell metaclones
Source: Nat Commun. 2026 Jan 21;17:1900. doi: 10.1038/s41467-026-68678-9 (PMC12923891; doi:10.1038/s41467-026-68678-9)
Supplement: Supplementary file 12 — Reporting Summary [file 41467_2026_68678_MOESM12_ESM.pdf]

nature portfolio

Corresponding author(s): Mahdad Noursadeghi

Last updated by author(s): Jan 10, 2026

Reporting Summary

Nature Portfolio wishes to improve the reproducibility of the work that we publish. This form provides structure for consistency and transparency in reporting. For further information on Nature Portfolio policies, see our Editorial Policies and the Editorial Policy Checklist.

Statistics

For all statistical analyses, confirm that the following items are present in the figure legend, table legend, main text, or Methods section.

n/a	Confirmed	
☐	☒	The exact sample size (*n*) for each experimental group/condition, given as a discrete number and unit of measurement
☐	☒	A statement on whether measurements were taken from distinct samples or whether the same sample was measured repeatedly
☐	☒	The statistical test(s) used AND whether they are one- or two-sided<br>*Only common tests should be described solely by name; describe more complex techniques in the Methods section.*
☒	☐	A description of all covariates tested
☐	☒	A description of any assumptions or corrections, such as tests of normality and adjustment for multiple comparisons
☐	☒	A full description of the statistical parameters including central tendency (e.g. means) or other basic estimates (e.g. regression coefficient) AND variation (e.g. standard deviation) or associated estimates of uncertainty (e.g. confidence intervals)
☐	☒	For null hypothesis testing, the test statistic (e.g. *F*, *t*, *r*) with confidence intervals, effect sizes, degrees of freedom and *P* value noted<br>*Give P values as exact values whenever suitable.*
☒	☐	For Bayesian analysis, information on the choice of priors and Markov chain Monte Carlo settings
☒	☐	For hierarchical and complex designs, identification of the appropriate level for tests and full reporting of outcomes
☐	☒	Estimates of effect sizes (e.g. Cohen's *d*, Pearson's *r*), indicating how they were calculated

Our web collection on statistics for biologists contains articles on many of the points above.

Software and code

Policy information about availability of computer code

Data collection

- REDCap

Data analysis

Custom code:
- Analysis code to reproduce the results and figures presented in the manuscript is available at https://github.com/carolinturner/tst_tcr (https://doi.org/10.5281/zenodo.18209647)
- Library code for Metaclonotypist is available at https://github.com/qimmuno/metaclonotypist (https://doi.org/10.5281/zenodo.17977729)

Commercial/open source software:
- Mapping of bulk RNAseq data: Kallisto v0.46
- Upstream regulator analysis: Ingenuity Pathway Analysis
- Network visualisation: Gephi v0.9.4
- TCR quantification: Decombinator v4
- TCR clustering: GLIPH version2 and Metaclonotypist
- Processing of published single cell TCRseq data: 10x Genomics CellRanger v7.1.0
- Assembly of figures: Inkscape v0.92
- Analysis in R (v 4.3.3), and python (v 3.10.4) via jupyter notebook (v7.2.1)
- Specific R packages: tximport (summing transcript-level counts on gene level); BioMart (gene annotation); SARtools v1.8.1 and DeSeq2 v1.42.1 (differential gene expression analysis); XGR v1.1.9 (Reactome pathway enrichment); ineq v0.2-13 and entropy v1.3.2 (TCR repertoire diversity); rstatix v0.7.2 (Wilcoxon tests); stats v4.3.3 (odds ratio calculations); pheatmap v1.0.12 and ComplexHeatmap v2.18.0 (heatmap visualisations); tidyverse v2.0.0 (data wrangling and visualisation); ggpubr v0.6.0 (assembling figures)

- Specific python packages: pyrepseq (convergence analysis and TCR sequence logos); igraph (metaclone visualisation); metaclonotypist (metaclone discovery)

For manuscripts utilizing custom algorithms or software that are central to the research but not yet described in published literature, software must be made available to editors and reviewers. We strongly encourage code deposition in a community repository (e.g. GitHub). See the Nature Portfolio guidelines for submitting code & software for further information.

Data

Policy information about availability of data

All manuscripts must include a data availability statement. This statement should provide the following information, where applicable:

- Accession codes, unique identifiers, or web links for publicly available datasets
- A description of any restrictions on data availability
- For clinical datasets or third party data, please ensure that the statement adheres to our policy

All source data for the analyses presented in this study are provided in the Source Data file. The processed RNAseq data generated in this study are available at Array Express with accession number E-MTAB-14687 [https://www.ebi.ac.uk/biostudies/ArrayExpress/studies/E-MTAB-14687?query=E-MTAB-14687%20]. The raw RNA sequencing data in FASTQ format are available under controlled access to comply with data privacy restrictions. Access can be obtained via the European Genome-Phenome Archive with accession number EGAD50000001208 [https://ega-archive.org/datasets/EGAD50000001208]. Data will be shared with investigators whose proposed use is within the scope of participant consent subject to a data access agreement. The processed TCR sequencing data generated in this study are available from UCL's Research Data Repository [https://doi.org/10.5522/04/28049606]. The raw TCR sequencing data in FASTQ format are available at NCBI Short Read Archive with accession number PRJNA1208718 [https://www.ncbi.nlm.nih.gov/bioproject/PRJNA1208718]. Previously published single-cell TCR sequencing data from human lung are available from Gene Expression Omnibus with accession numbers GSE253828 [https://www.ncbi.nlm.nih.gov/geo/query/acc.cgi?acc=GSE253828] (TB patients) and GSE154826 [https://www.ncbi.nlm.nih.gov/geo/query/acc.cgi?acc=GSE154826] (lung cancer patients).

Research involving human participants, their data, or biological material

Policy information about studies with human participants or human data. See also policy information about sex, gender (identity/presentation), and sexual orientation and race, ethnicity and racism.

Reporting on sex and gender	Male and female participants were included in this study. Sex was determined by self-reporting. Overall numbers are reported in Tables 1 and 2 of the manuscript. The primary analysis included broadly equal proportions of male and female sex. The analyses were not disaggregated by sex because this was not a component of the research question.
Reporting on race, ethnicity, or other socially relevant groupings	Ethnicity was derived from self-reported country of birth, categorised into commonly used genetic ancestry groups.
Population characteristics	Study participants comprised healthy HIV seronegative adults with immune memory for Mtb-specific antigens identified by positive peripheral blood IFN-gamma release assays using the QuantiFERON Gold Plus Test, but no clinical or radiological evidence of active tuberculosis. Summary-level participant characteristics (sex, age, ethnicity) are reported in Tables 1 and 2 of the manuscript.
Recruitment	Consecutive eligible adult patients attending North Central London Cluster of TB clinics for assessment following positive screening Quantiferon Gold Plus Test result were invited to participate by providing written information on participation in the study, approved by he UK Human Research Authority. The summary demographic characteristics of the study population was comparable to that of the whole clinic population in terms of age, sex and ethnicity, with no evidence of participant selection bias.
Ethics oversight	Research ethics and regulatory approvals for the present study were provided by UK National Research Ethics Service (NRES) Committee (Fulham) reference nos 11/LO/1863 and 18/LO/0680, and the NRES Committee (Camden and Islington) reference no 14/LO/0505.

Note that full information on the approval of the study protocol must also be provided in the manuscript.

Field-specific reporting

Please select the one below that is the best fit for your research. If you are not sure, read the appropriate sections before making your selection.

[x] Life sciences [ ] Behavioural & social sciences [ ] Ecological, evolutionary & environmental sciences

For a reference copy of the document with all sections, see nature.com/documents/nr-reporting-summary-flat.pdf

Life sciences study design

All studies must disclose on these points even when the disclosure is negative.

Sample size	The analysis presented did not form the basis of sample size calculations. The sample size for the primary analysis in the present manuscript was determined by the availability of tuberculin skin test RNA samples from the study cohort.
Data exclusions	All available data were included in the RNAseq analysis.<br>For TCRseq analyses, the primary analysis (presented in the main manuscript) excludes samples with <16,000 reads and reports findings in down-sampled (equal-sized) repertoires. The secondary analysis (presented in the supplementary material) confirms findings in full

	repertoires of all samples. The threshold of 16,000 reads was chosen as smallest repertoire in an initial subset of the full dataset.<br>For metaclone discovery, samples with <5,000 reads and no available HLA data were excluded. The threshold of 5,000 reads was chosen to retain the maximum number of samples while reducing uneven sampling.<br>All data exclusions are also detailed in the consort diagram in Supplementary Figure 1.
Replication	All analyses included data from multiple participants. Results were reported with 95% confidence intervals. The key finding of generaliseable Mtb-reactive T cell metaclones was validated by showing their enrichment in multiple independent data sets. All performed experiments were included.
Randomization	Randomization was not relevant because the main analysis was a group-wise comparison of timepoints across all participants.
Blinding	Sample processing was blinded to metadata and group allocations. For analysis, blinding was not relevant because group-wise comparisons were predominantly made between timepoints across all participants.

Reporting for specific materials, systems and methods

We require information from authors about some types of materials, experimental systems and methods used in many studies. Here, indicate whether each material, system or method listed is relevant to your study. If you are not sure if a list item applies to your research, read the appropriate section before selecting a response.

Materials & experimental systems

n/a	Involved in the study
☒	☐ Antibodies
☒	☐ Eukaryotic cell lines
☒	☐ Palaeontology and archaeology
☒	☐ Animals and other organisms
☒	☐ Clinical data
☒	☐ Dual use research of concern
☒	☐ Plants

Methods

n/a	Involved in the study
☒	☐ ChIP-seq
☒	☐ Flow cytometry
☒	☐ MRI-based neuroimaging

Plants

Seed stocks	*Report on the source of all seed stocks or other plant material used. If applicable, state the seed stock centre and catalogue number. If plant specimens were collected from the field, describe the collection location, date and sampling procedures.*
Novel plant genotypes	*Describe the methods by which all novel plant genotypes were produced. This includes those generated by transgenic approaches, gene editing, chemical/radiation-based mutagenesis and hybridization. For transgenic lines, describe the transformation method, the number of independent lines analyzed and the generation upon which experiments were performed. For gene-edited lines, describe the editor used, the endogenous sequence targeted for editing, the targeting guide RNA sequence (if applicable) and how the editor was applied.*
Authentication	*Describe any authentication procedures for each seed stock used or novel genotype generated. Describe any experiments used to assess the effect of a mutation and, where applicable, how potential secondary effects (e.g. second site T-DNA insertions, mosiacism, off-target gene editing) were examined.*
